# Emergency Departments in Contemporary Healthcare: Are They Still for Emergencies? An Analysis of over 1 Million Attendances

**DOI:** 10.3390/healthcare12232426

**Published:** 2024-12-03

**Authors:** Arian Zaboli, Gianni Turcato, Gloria Brigiari, Magdalena Massar, Marta Ziller, Serena Sibilio, Francesco Brigo

**Affiliations:** 1 Innovation, Research and Teaching Service (SABES-ASDAA), Teaching Hospital of the Paracelsus Medical Private University (PMU), 39100 Bolzano, Italy; zaboliarian@gmail.com (A.Z.); magdalena.massar@sabes.it (M.M.); 2 Department of Internal Medicine, Intermediate Care Unit, Hospital Alto Vicentino (AULSS-7), 36014 Santorso, Italy; gianni.turcato@yahoo.it; 3 Unit of Biostatistics, Epidemiology and Public Health, Department of Cardiac, Thoracic, Vascular Sciences and Public Health, University of Padova, 35129 Padova, Italy; gloria.brigiari@ubep.unipd.it; 4 Cardiology Department, Hospital of Bolzano, 39100 Bolzano, Italy; marta.ziller@sabes.it; 5 Department Public Health, Institute of Nursing Science, Universitat Basel, 4051 Basel, Switzerland; serena.sibilio@unibas.ch

**Keywords:** emergency department, non-urgent care, urgent care, workload, triage, health policy, health services needs and demand

## Abstract

Background: Over the past few decades, emergency departments (EDs) have experienced an increasing workload. However, the variation in the types of patient accesses to these departments remains poorly understood. Objective: To evaluate the 5-year temporal trend in the volume of patients attending EDs based on the urgency of their conditions. Methods: This multicenter observational retrospective study was conducted from 1 January 2019, to 31 December 2023, across seven Italian EDs located within the same province. All patients accessing the EDs during the study period were included, totaling 1,282,735 patients. The triage code was used as an urgency index; non-urgent patients were defined as those who received a code 4 or 5 in triage, while urgent patients were defined as those who received a code 3, 2, or 1 in triage. Temporal analyses of admissions were conducted, also evaluating individual age groups to understand behavior over time. Results: From 2019 to 2023, there was a significant 10% increase in ED attendances by non-urgent patients. This increase was observed during both daytime and nighttime shifts. Notably, all age groups showed an increase in non-urgent patients, except for pediatric patients aged 0 to 14. Conclusions: Over the past 5 years, there has been a consistent upward trend in ED attendances by non-urgent patients. Healthcare policies should consider implementing strategies to manage or mitigate the overload in EDs, particularly related to non-urgent patient accesses.

## 1. Introduction

Over the past few decades, there has been a significant increase in the number of non-urgent patients presenting to emergency departments (EDs) [1,2]. This trend has been exacerbated by the COVID-19 pandemic, which strained healthcare resources and altered patient behaviors [3,4,5]. Multiple factors contribute to this rise, including prolonged waiting times for outpatient visits for minor issues, prompting patients to seek faster care in EDs [6,7,8]. The exact causes of this trend remain not entirely clear [3]. However, changes in access to community healthcare services could have played a relevant role. For example, there has been a decline in face-to-face consultations, stricter requirements for primary care appointments, and limited availability of timely care [4,6]. These challenges may drive patients to seek care at EDs, which provide 24/7 access to medical care, even if this involves longer wait times [7,8]. Additionally, EDs often feature advanced diagnostic resources that are not typically accessible in community healthcare settings, making them an attractive option for patients seeking comprehensive diagnostic services, even for non-urgent health concerns [3,4,7,8]. This growing reliance on EDs for non-urgent cases has significantly added to the burden on emergency healthcare systems.

In response to these challenges, EDs have developed multiple pathways over the past decade to redirect non-urgent patients to other care areas within the same facility [9,10,11]. One widely adopted strategy is the integration of general practitioners (GPs) within the ED to manage less severe cases [9]. Additional options include Urgent Treatment Centers (UTCs), which focus on managing minor injuries and issues [10], and fast-track pathways that refer patients with minor problems directly to specialist clinics from the ED [11,12].

While these strategies effectively manage patient flow and alleviate ED congestion, they may also inadvertently encourage patients to access EDs due to the promise of quicker treatment and shorter wait times compared to scheduling non-urgent specialist visits [7,8,13]. This study aims to evaluate the temporal trend in the volume of both urgent and non-urgent patients attending EDs.

## 2. Methods

### 2.1. Study Design and Setting

This multicenter observational retrospective study was conducted from 1 January 2019 to 31 December 2023 across seven emergency departments (EDs) in hospitals located in the Italian province of South Tyrol, Northern Italy. The participating EDs included those in Bolzano, Merano, Bressanone, Brunico, Silandro, Vipiteno, and San Candido. The hospitals included in this study are all part of the provincial healthcare network.

The main hospital, located in Bolzano, serves as the central facility and offers specialized services, including emergency cardiology, neurosurgery, and a trauma center. Merano Hospital, the second largest in the province, provides a broad range of medical services but is not equipped to manage the full spectrum of cases handled by the main hospital. Bressanone and Brunico hospitals are mid-sized facilities situated in mountainous areas, where patient volumes fluctuate significantly due to alpine tourism. While these hospitals manage a similar patient profile to Merano Hospital, they operate on a smaller scale due to lower overall patient numbers. In contrast, Silandro, Vipiteno, and San Candido are smaller rural hospitals focused on serving the needs of their local communities. Although these facilities are equipped to handle acute cases, patients requiring more advanced or urgent care are transferred to the nearest larger hospitals. Furthermore, these rural hospitals operate with reduced capacity during nighttime hours, with limited availability of laboratory and radiology services.

In all these EDs, triage assessments are performed by specially trained nurses utilizing the Manchester Triage System (MTS) [14]. The MTS is globally recognized as one of the most widely used and validated triage systems. It categorizes patients into five priority levels: priority 5 (blue, non-urgent), priority 4 (green, standard), priority 3 (yellow, urgent), priority 2 (orange, very urgent), and priority 1 (red, emergency). This standardized triage system has been uniformly adopted across all participating EDs since 2016, ensuring consistency in patient assessment and categorization.

### 2.2. Data Collection

Data were sourced from mandatory annual reports submitted to the Italian Ministry of Health. These reports include comprehensive information on patient visits, including demographics, triage categories, diagnoses, and outcomes. Data were collected from the electronic health records (EHRs) of patients attending the seven EDs during the study period. The following variables were included:

(a) Patient age; (b) Triage code; (c) Time from patient admission to medical assessment; (d) Time from patient admission to the end of the ED visit; (e) Outcome after the ED visit, categorized as: 1. Discharge; 2. Admission to the hospital; 3. Left ED before initial medical assessment; Left ED after medical assessment but before discharge; 4. Other outcomes, including: death in the ED; refusal of hospital admission; patients brought in by emergency services but already deceased; transfer to another institution outside the seven hospitals; (f) Readmission to the ED in the following 24 h.

These variables were chosen to provide a comprehensive overview of patient flow and outcomes within the EDs, allowing for a detailed analysis of both urgent and non-urgent patient visits.

All patients who accessed the ED and had complete records for the aforementioned variables were included in the study.

### 2.3. Standardization of Admissions

To evaluate the differences in ED admissions over the years, patients with priority codes 4 and 5 (blue and green according to MTS) were compared against those with priority codes 3, 2, and 1 (yellow, orange, and red according to MTS). Patients with priority codes 4 and 5 were classified as non-urgent, while those with priority codes 3, 2, and 1 were classified as urgent.

Given the variability in admissions during the pandemic period, standardization was applied. The percentage of patients admitted each month was assessed using the following formula:

$$Percentage\;of\;non-urgent\;patients\;per\;month=\frac{number\;of\;priority\;4\;and\;5\;per\;month\times 100}{total\;admission\;per\;month}$$

$$Percentage\;of\;urgent\;patients\;per\;month=\frac{number\;of\;priority\;3,\;2,\;and\;1\;per\;month\times 100}{total\;admission\;per\;month}$$

This approach enabled the calculation of the monthly percentage of urgent and non-urgent patients arriving in the ED, independent of the total number of patients. Additionally, in a sub-analysis, data were divided between the daytime shift (08:00–20:00) and the nighttime shift (20:00–08:00), with proportions calculated based on the total number of patients admitted each month. The decision to structure the timings in this manner was based on the alignment with the 12-h shift schedules of the ED staff working in the facilities under study.

To determine if there were differences among various age groups, patients were categorized into four groups: pediatric (0 to 14 years old), young adults (15 to 35 years old), adults (36 to 65 years old), and elderly (66 years old and above).

### 2.4. Statistical Analysis

Continuous variables were summarized using medians with interquartile ranges (IQR). Categorical variables were expressed as percentages and total counts.

Subsequently, distribution graphs were generated to analyze month-by-month data over the 5-year period, comparing the proportions of urgent and non-urgent patients throughout the study. We also created distribution graphs by dividing the data into daytime (08:00–20:00) and nighttime (20:00–08:00) accesses. These graphs allowed us to evaluate variations related to patient age groups and the timing of their access.

To estimate the underlying trend, we applied Generalized Additive Models (GAM), a flexible class of regression models that extend linear models by allowing non-linear relationships between the predictors and the response variable [15]. In our analysis, we use a GAM to model the temporal trend in percentage of ED accesses. The underlying function is expressed as:

y = β0 + s(x) + ϵ

where y represents the percentage of ED accesses, β0 is the intercept, s(x) is a smooth function of the predictor x (month of ED access), and ϵ is the error term. The smooth function s(x) is modeled using cubic regression splines, which capture complex, non-linear patterns in the data, providing a robust representation of the trend over time. We employ the restricted maximum likelihood (REML) method to estimate the model parameters, ensuring robust and reliable smoothing [16].

In the analysis of time series data, particularly within complex contexts featuring non-linear patterns over time, the GAM framework enables the modeling of non-linear relationships without requiring a strict parametric form, providing a more accurate representation than traditional linear models. Additionally, in this case, the GAM method was applied exclusively for descriptive purposes and not for conducting comparative analysis. All statistical analyses were performed using R version 4.3.3.

### 2.5. Ethical Consideration

Ethical approval for this study was obtained from the Institutional Review Board (IRB) of the Ethical Committee for Clinical Trials of the Autonomous Province of Bolzano (approval number: 28-2024). Data-anonymization procedures were implemented to safeguard participant confidentiality and privacy. Personally identifiable information was removed or replaced with unique identifiers to prevent the identification of individual participants. This study was conducted in accordance with the ethical standards outlined in the Declaration of Helsinki and other relevant international guidelines for biomedical research involving human subjects.

## 3. Results

During the study period, a total of 1,282,735 patients were included. The distribution of patients by year and by hospital is shown in Table 1.

The characteristics of the enrolled patients are summarized in Table 2. The mean age of the patients was 45 years (IQR 23–66). Priority 4 was the most frequently assigned triage code, accounting for 62.0% (779,391 out of 1,282,735) of the patients. The median time from ED arrival to medical evaluation was 24 min (IQR 9–54), while the median time from ED arrival to discharge or admission was 102 min (IQR 55–184). After evaluation in the ED, 85% of patients (1,066,420 out of 1,282,735) were discharged, while 11.0% (143,517 out of 1,282,735) required hospitalization (Table 2). Finally, only 2.5% (32,194 out of 1,282,735) of the patients returned to the ED within 24 h of their previous discharge.

**Figure 1:**
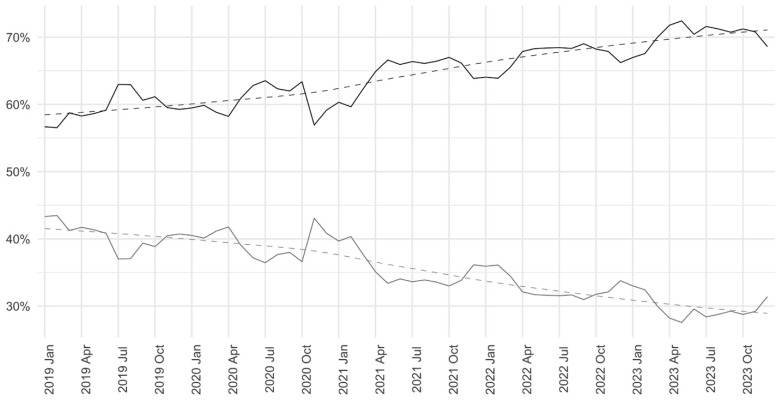
Percentage of non-urgent patients (triage code 4 and 5) over the years compared to patients with urgent code (triage code 3, 2, and 1). The black line represents non-urgent patients while the gray line represents urgent patients. The dotted line represents the temporal trend estimated directly from the data (for details about the model and the smoothing parameter used see the Methods section).

Figure 1 illustrates the monthly proportions of ED visits, distinguishing between non-urgent patients (priority 4 and 5) and urgent patients (priority 1, 2, and 3), revealing a marked increase in non-urgent patients compared to urgent patients. During the period from 2019 to 2023, there was an approximate 10% increase in the number of non-urgent patients on total ED attendances, while the number of urgent patients decreased by about 10%.

**Figure 2:**
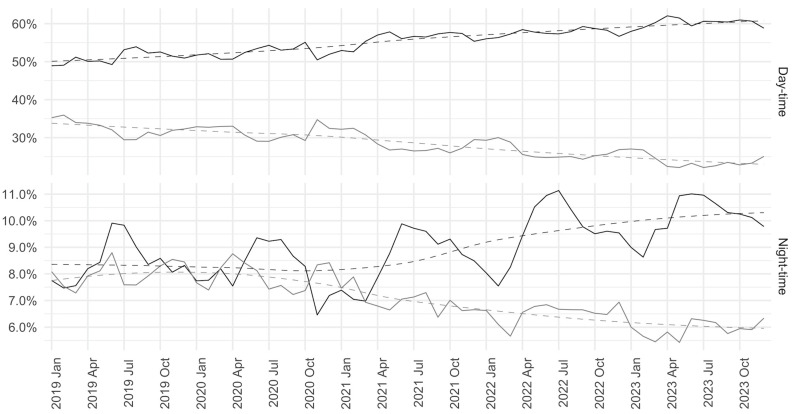
Proportion of patients accessing with non-urgent codes (code 4 and 5) and urgent codes (code 3, 2, and 1) over the years, distinguishing daytime accesses (8:00–20:00) and nighttime accesses (20:00–8:00). The black line represents non-urgent patients while the gray line represents urgent patients. The dotted line represents the temporal trend estimated directly from the data (for details about the model and the smoothing parameter used see the Methods section).

Over the study period there was an increase in the proportion of non-urgent patients both in the day and night shifts (Figure 2).

**Figure 3:**
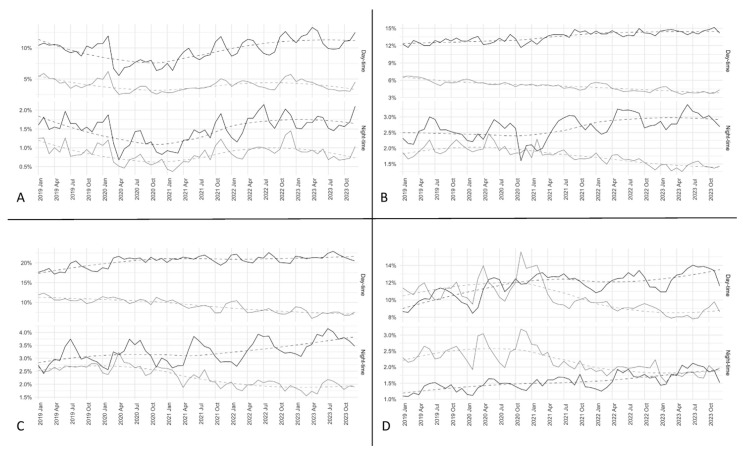
Proportion of non-urgent patients (triage code 4 and 5) and urgent codes (triage code 3, 2, and 1) accessing over the years, distinguishing daytime and nighttime accesses. The black line represents non-urgent patients while the gray line represents urgent patients. In (**A**), only patients aged 0 to 14 years are reported, while in (**B**), only patients aged 15 to 30 years are reported. In (**C**), only patients aged 31 to 65 years are reported, while in (**D**), only patients aged > 65 years are reported. The dotted line represents the temporal trend estimated directly from the data (for details about the model and the smoothing parameter used see the Methods section).

In ED attendances of pediatric patients, the proportion of non-urgent daytime visits on total ED accesses has remained stable from 2019 to 2023 (Figure 3). In contrast, pediatric urgent ED attendances have decreased since 2019, both during daytime and nighttime shifts (Figure 3A).

For young adults (i.e., individuals aged 15–30 years), a notable divergence between urgent and non-urgent patients has emerged over the years, evident in both daytime and nighttime shifts (see Figure 3B). In contrast, for adult patients (31–65 years), there has been an increase in non-urgent visits in both daytime and nighttime shifts, alongside a decline in urgent visits (see Figure 3C).

Conversely, elderly patients (>65 years) have witnessed a reversal in trend during daytime shifts (see Figure 3D). In 2019, they constituted the only category with more urgent visits than non-urgent ones; however, this pattern inverted in 2020, with a rise in non-urgent patients. During nighttime shifts, the urgent and non-urgent ED attendances of elderly patients stabilized at similar levels. Nevertheless, in 2019, elderly patients attending EDs were predominantly urgent cases, while by 2023, urgent cases in this age group had decreased to levels similar to those of non-urgent cases.

## 4. Discussion

The present study has revealed significant shifts in the temporal patterns of patient volume seeking assistance in EDs, indicating an increase in non-urgent cases relative to urgent ones.

These findings have critical implications for clinical practice. In recent years, EDs have experienced substantial changes in the demographics of patients they serve, characterized by a consistent increase in non-urgent cases [3,4,5,9]. This trend may stem from various factors, with ED accessibility playing a pivotal role [13,17]. Operating 24/7, EDs offer patients unrestricted access, unlike the increasingly appointment-dependent nature of primary care, which often leads to prolonged and impractical wait times, prompting patients to resort to EDs [6,18,19]. Moreover, while the evolution of EDs has introduced specialized pathways and alternatives for managing patient influx, it paradoxically fosters patient reliance on EDs over seeking alternative care avenues [13,20]. The study does not aim to suggest that patients with codes 4 or 5 should not come to the ED, but rather to demonstrate that there has been a significant change in the type of patients arriving at the ED. The ED was originally designed primarily for the management of acute and life-threatening issues that cannot wait to be evaluated by other physicians through different means. However, the study has shown that EDs are facing a growing influx of low-priority patients, which is impacting the care of urgent and time-sensitive patients, whose numbers have decreased over the years.

Health policies must acknowledge these profound changes and respond accordingly, either by restricting access to EDs to urgent cases or by enhancing ED infrastructure to accommodate the evolving patient load. This necessitates a recalibration of healthcare strategies, potentially prioritizing ED access while reevaluating the emphasis on primary care—thereby strengthening ED capabilities. Despite significant funding and territorial improvements in recent years, EDs continue to experience increasing visitation rates, suggesting a preference among patients for the comprehensive resources available within EDs compared to the relatively limited resources in community healthcare settings [13,21,22].

The reasons behind this trend remain somewhat unclear. Over time, changes in territorial healthcare have introduced significant barriers to primary care access. For instance, the growing reliance on scheduled appointments with general practitioners, as opposed to spontaneous visits, often leads to delays, potentially prompting patients to seek care at EDs, where no appointment is required [4,6]. EDs also offer immediate evaluations and access to diagnostic tools that may not be readily available in community healthcare settings.

Additionally, the COVID-19 pandemic has contributed to a notable reduction in face-to-face consultations, a mode of care that has not fully recovered post-pandemic, thereby creating access challenges for certain segments of the population [6]. Moreover, Italy’s universal, free healthcare system imposes no access restrictions on patients, which may further encourage higher ED utilization, including for cases that might be more appropriately managed outside the ED [3,4].

Furthermore, the decline in nighttime visits for urgent issues suggests that EDs are primarily managing non-urgent cases during these hours. This data holds significant implications not only for patients but also for healthcare staff working within these facilities. It has long been recognized that nighttime shifts are not only physically demanding but also pose substantial health risks to workers, including increased mortality and cancer risks compared to those who do not work night shifts [23,24]. Thus, healthcare policies should address the issue of nighttime visits, either by informing and raising awareness among the population or by implementing measures to limit such visits and redirect patients to daytime care. Some countries, such as Finland, have begun exploring these strategies and alternatives to promote the appropriate utilization of EDs and alleviate the workload for healthcare professionals during nighttime hours [25,26]. A recent Finnish study discovered that 73.5% of non-urgent patients who presented at night did not undergo any investigative procedures, suggesting the feasibility of alternative care pathways for this patient cohort [25]. Furthermore, Paulin et al. previously emphasized the need to restructure EDs to address the inappropriate utilization by non-urgent patients. Their study, involving 40,263 patients, revealed that 46.0% of them had a National Early Warning Score 2 (NEWS-2) score of 0, indicating a low level of urgency [26].

Excluding the pediatric population (0–14 years old), all other patient categories in our study have witnessed a proportional increase in non-urgent cases and a decline in urgent ones, underscoring the critical need to reassess the priorities and operations of EDs. In recent years, there has been a noticeable rise in Length of Stay (LOS) within EDs, prompting the adoption of the 4-h target for patient discharge or admission [27,28]. However, this prolonged LOS is often attributed to the higher presence of non-urgent patients who, in overcrowded ED settings, endure longer wait times before receiving attention [29]. This 4-h target should be approached with caution, considering that amidst the persistent influx of non-urgent patients, achieving expedited patient discharges presents significant challenges [28,29].

In response to this scenario, there has been a growing allocation of resources towards establishing tailored pathways for specific patient demographics, particularly targeting the frail or elderly [30]. Nonetheless, a more focused approach on developing discharge criteria or discouraging ED access for minor conditions could have potentially obviated the necessity for such specialized pathways [31,32,33]. This underscores a significant challenge, demanding healthcare strategies to adeptly redirect patients to alternative facilities while devising efficient tools for prompt discharge, either at triage or during initial assessment [31,32,33]. Such initiatives could be crucial for mitigating the burden on EDs and optimizing patient care pathways.

From a workforce perspective, it is apparent that fewer individuals are inclined to pursue employment in EDs, primarily attributed to the congestion and persistent exposure to risks and overwhelming workloads [34,35]. Therefore, it is imperative to investigate and evaluate secure models for expeditious patient discharge, facilitating their transition to alternative healthcare services.

This retrospective study has a few limitations. Firstly, the assignment of the priority code was not independently verified, which introduces some uncertainty regarding the strict application of the MTS. Consequently, there may be patients within the categorized groups who were triaged incorrectly. However, in all our EDs, triage nurses undergo annual audits to monitor their performance, and the triage execution approach remained unchanged throughout the 5-year study period. Secondly, the COVID-19 pandemic affected the results by significantly altering the number of ED visits across all facilities under study, resulting in an overall reduction in visits during this period. However, given the long observation period and the standardization measures applied, the impact of the pandemic is likely to have been time limited. Excluding this period would not be appropriate, as the pandemic itself may have influenced patient behavior, prompting more frequent visits to the ED. Thirdly, while data on ED diagnoses were available, they were not utilized due to the lack of standardized collection methods. This lack of standardization made it unfeasible to group diagnoses effectively across such a large sample.

## 5. Conclusions

This study has revealed a marked increase in the proportion of non-urgent patients visiting the ED over the past 5 years, accompanied by a decline in urgent cases. Furthermore, while pediatric patients have maintained a stable balance between urgent and non-urgent cases during this period, there has been an increase in non-urgent visits among young adults, adults, and the elderly.

Healthcare policies must tackle this issue through practical, targeted solutions aimed at redirecting non-urgent patients away from the ED. Potential measures include expanding the operating hours of primary care facilities to offer viable alternative care options and introducing a non-urgent referral system tailored to specific patient groups, such as certain age demographics or non-emergency cases.

By implementing these targeted actions, patient care within the ED could be improved, with reduced wait times and a lighter workload for ED healthcare personnel. These measures would contribute to the development of a more efficient, sustainable, and patient-centered emergency healthcare system.

## Figures and Tables

**Table 1:** Distribution of Included Patients by Year and Hospital.

	Overall	2019	2020	2021	2022	2023
All hospitals	1.282.735	279.960	201.433	221.642	279.642	279.717
Bolzano hospital, n (%)	407.357 (32.0)	87.435 (31.0)	65.455 (32.0)	72.562 (33.0)	86.828 (31.0)	95.087 (32.0)
Merano hospital, n (%)	302.895 (24.0)	68.625 (25.0)	46.436 (23.0)	53.842 (24.0)	66.465 (24.0)	67.527 (23.0)
Bressanone hospital, n (%)	180.884 (14.0)	38.600 (14.0)	28.956 (14.0)	31.140 (14.0)	39.583 (14.0)	42.605 (14.0)
Brunico hospital, n (%)	179.897 (14.0)	39.055 (14.0)	27.234 (14.0)	28.690 (13.0)	39.577 (14.0)	45.341 (15.0)
Silandro hospital, n (%)	90.969 (7.1)	19.387 (6.9)	13.995 (6.9)	15.852 (7.2)	20.733 (7.4)	21.002 (7.0)
San Candido hospital, n (%)	63.146 (4.9)	14.703 (5.3)	9.685 (4.8)	9.685 (4.8)	13.609 (4.9)	15.437 (5.1)
Vipiteno hospital, n (%)	57.577 (4.5)	12.155 (4.3)	9.672 (4.8)	9.672 (4.4)	12.922 (4.6)	12.984 (4.3)

**Table 2:** Patient Characteristics by Year of Inclusion.

	Overall	2019	2020	2021	2022	2023
Age, years, mean (SD)	45 (26)	44 (26)	46 (26)	45 (26)	44 (26)	44 (26)
Triage priority code, n (%)						
Priority code 1	10.962 (0.9)	2.016 (0.7)	1.685 (0.8)	1.784 (0.8)	2.877 (1.1)	2.600 (0.9)
Priority code 2	85.995 (6.7)	21.897 (7.8)	17.175 (8.5)	16.375 (7.4)	15.609 (5.6)	14.939 (5.0)
Priority code 3	333.365 (26.0)	82.561 (29.5)	56.837 (28.2)	57.356 (25.9)	69.419 (24.8)	67.192 (22.4)
Priority code 4	779.391 (60.7)	157.675 (56.3)	115.987 (57.6)	135.210 (61.0)	174.633 (62.4)	195.886 (65.3)
Priority code 5	52.946 (4.1)	9.062 (3.3)	6.669 (3.3)	8.920 (4.0)	13.466 (4.8)	14.829 (4.9)
Left before triage	20.075 (1.6)	6749 (2.4)	3080 (1.6)	1996 (0.9)	3713 (1.3)	4537 (1.5)
Time from admission to initial medical assessment, minutes, median (IQR)	24 (9–54)	24 (10–56)	21 (9–47)	27 (12–55)	23 (10–52)	24 (8–60)
Priority code 1	5 (2–10)	7 (4–11)	8 (4–13)	7 (4–13)	4 (2–8)	2 (1–5)
Priority code 2	15 (6–29)	17 (7–33)	17 (7–32)	18 (8–32)	13 (6–23)	10 (3–22)
Priority code 3	19 (8–43)	21 (8–49)	19 (8–49)	24 (11–49)	18 (8–38)	16 (9–39)
Priority code 4	27 (11–61)	28 (11–63)	23 (9–51)	29 (13–61)	27 (11–58)	29 (10–67)
Priority code 5	38 (15–87)	30 (10–74)	29 (11–63)	37 (16–77)	43 (17–97)	46 (17–106)
Time from admission to the end of ED visit, minutes, median (IQR)	102 (55–184)	99 (52–178)	99 (52–180)	101 (55–183)	104 (57–184)	106 (58–191)
Priority code 1	123 (66–225)	98 (55–179)	142 (76–256)	143 (75–245)	139 (80–236)	102 (53–208)
Priority code 2	159 (92–285)	148 (86–262)	171 (98–316)	173 (100–308)	160 (96–280)	146 (83–260)
Priority code 3	125 (69–229)	120 (64–222)	125 (69–232)	127 (71–234)	127 (71–230)	126 (70–231)
Priority code 4	90 (49–158)	85 (46–150)	81 (43–145)	87 (48–152)	92 (51–160)	98 (53–174)
Priority code 5	95 (49–173)	88 (43–166)	80 (41–147)	85 (46–150)	101 (54–177)	102 (53–208)
Outcome, n (%)						
Discharge	1.066.420 (85.0)	230.467 (84.0)	163.840 (83.0)	183.790 (84.0)	236.411 (86.0)	251.912 (85.0)
Admission to the hospital	143.517 (11.0)	31.562 (12.0)	26.616 (13.0)	27.565 (13.0)	28.707 (10.0)	29.067 (9.9)
Left ED before medical assessment	21.804 (1.7)	5.290 (1.9)	2.906 (1.5)	3.227 (1.5)	4.808 (1.7)	5.573 (1.9)
Left ED after medical assessment	12.643 (1.0)	2.501 (0.9)	2.347 (1.2)	2.000 (0.9)	2.577 (0.9)	3.218 (1.1)
Other						
Readmission to the ED within 24 h, n (%)	32.194 (2.5)	5.812 (2.1)	4.678 (2.3)	5.902 (2.7)	6.945 (2.5)	8.857 (3.0)

## Data Availability

Data available on request due to privacy/ethical restrictions.
